# Apoptotic microparticles generated during acute HIV-1 infection inhibit human dendritic cells via CD44

**DOI:** 10.1186/1742-4690-9-S2-P183

**Published:** 2012-09-13

**Authors:** D Frleta, CE Ochoa, HB Kramer, SA Khan, AR Stacey, P Borrow, BM Kessler, BF Haynes, N Bhardwaj

**Affiliations:** 1 New York University Langone Medical Center, New York, NY, USA; 2 University of Oxford, Oxford, UK; 3 Duke University Medical Center, Durham, NC, USA

## Background

Acute human immunodeficiency virus type 1 (HIV-1) infection results in dysregulated immunity which contributes to poor control of viral infection. Dendritic cells (DCs) are key regulators of both adaptive and innate immune responses needed for controlling HIV-1 and we surmised that plasma factors elicited during acute HIV-1 infection (AHI) may impede DC function. Such inhibitory factors present in AHI plasma include apoptotic microparticles (MPs), small membranous blebs derived from dying cells.

## Methods

Plasma samples over sequential time points were obtained from AHI patients or healthy controls. Apoptotic MPs were isolated from supernatant of UV-irradiated PBMCs, AHI patient plasma or control plasma. Human DCs were treated with MPs or 10% plasma (control or AHI) and subsequently stimulated with various TLR agonists. DC activation was then assessed. MP-specific receptors were isolated from the DC surface and sequenced by mass spectrometry.

## Results

AHI plasma inhibited TLR-stimulated DC cytokine production. The inhibitory capacity of AHI plasma occurs at time of viral ramp-up, whereas plasma at times before plasma viremia is not inhibitory. We determined this inhibition was not mediated by virus. Because apoptotic MPs are elevated in AHI plasma, we treated DCs with experimental and AHI plasma-derived MPs, both of which reduced DC activation. The inhibition of DCs by AHI plasma or MPs blocked DC capacity to prime IFNg-producing T helper 1 CD4+ T cells as well as NK cell activation. Mass spectrometric analysis revealed CD44 a MP receptor, and blocking CD44 on DCs relieves MP-mediated suppression. Direct ligation of CD44 also inhibits DC activation. MP-CD44 interaction activates Rac1, c-Abl, and AKt signaling.

## Conclusion

Determining the factors in AHI which block DC function will provide potential targets to stimulate HIV-specific immunity. We are currently investigating molecular mechanisms of CD44-mediated DC inhibition and downstream signaling events that can be targeted to alleviate DC inhibition.

